# Unilateral Anatomical Variation of the Suprascapular and Dorsal Scapular Arteries: A Cadaveric Case Report

**DOI:** 10.7759/cureus.85095

**Published:** 2025-05-30

**Authors:** Lila Huston, Arunabh Bhattacharya

**Affiliations:** 1 Department of Applied Biomedical Sciences, University of the Incarnate Word School of Osteopathic Medicine, San Antonio, USA

**Keywords:** axillary artery, dorsal scapular artery, suprascapular artery, supraspinatus muscle, thyrocervical trunk

## Abstract

While most anatomical variations in cervical neurovasculature remain asymptomatic throughout life, clinical impacts may arise during surgical procedures, potentially worsening patient outcomes. This case report highlights two unique anatomical variations observed in the arteries of the right neck and shoulder regions of a 75-year-old Caucasian male donor body. The first variation was the origin of the suprascapular artery (SSA), the primary blood supply to two of the four rotator cuff muscles. The SSA arose directly from the first part of the axillary artery instead of the thyrocervical trunk off the subclavian artery (SA). The vessel passed over the lower trunk and continued obliquely between the contributions of the upper and middle trunks to the lateral cord of the brachial plexus before coursing posteriorly, where it divided further into two branches. While one branch continued to the supraspinous fossa, the second branch split into three smaller branches to supply the subscapularis muscle. The second variation concerned the dorsal scapular artery. The artery emerged normally from the third part of the SA but provided branches to the supraspinatus muscle in addition to its normal blood supply to the rhomboid and levator scapulae muscles. Knowledge of potential anatomical variations of the origin and distribution of the branches of the SA is crucial for surgeons, given the importance of this vasculature. Awareness and prior identification of variations such as those presented in this report can help avoid potential complications in surgical procedures.

## Introduction

The subclavian artery (SA) plays an important role in supplying blood to several key structures, including muscles of the superficial back and cervical region, and the entirety of the upper limb through its continuation as the axillary artery (AA), brachial artery, and ultimately the ulnar and radial arteries [1]. The anterior scalene muscle (ASM) divides the SA into three major subdivisions, which, through their specific branches, supply the cervical, shoulder, and pectoral regions, scalp, ribs, and the thyroid/parathyroid glands. The first section of the SA that lies medial to the ASM has three main branches: (1) the vertebral artery, which ascends and supplies the cervical vertebrae and their musculature, and ultimately portions of the brain; (2) the thyrocervical trunk (TCT), which branches into the inferior thyroid artery (ITA) that supplies the inferior lobe of the thyroid gland and some of the infrahyoid muscles, the suprascapular artery (SSA), which supplies the supraspinatus and infraspinatus muscles, and the transverse cervical artery (TCA), which supplies the trapezius muscle; and (3) the internal thoracic artery (ITHA), which descends inferiorly to supply the breasts, and anterior chest wall muscles - intercostals and transversus thoracis. The costocervical trunk emerges from the second section of the SA, deep to the ASM, and branches to the deep cervical and superior intercostal arteries. The deep cervical artery supplies muscles of the neck, and the superior intercostal artery descends inferiorly to supply the first set of intercostal muscles. The final section of the SA, which lies between the ASM and the first rib, has one branch, the dorsal scapular artery (DSA), which is the primary blood supply to the posterior back muscles - rhomboid major and minor, and levator scapulae.

While the branching and distribution of these vessels is generally consistent, anatomic variations based on different origins and branching patterns have been described in the literature [2-4]. For instance, SSA has been shown to arise independently from SA and AA [2-5]. Some studies also describe TCA, DSA, ITA, and ITHA as the origin of SSA [6-10], whereas two studies, including one from our group, reported the vessel to be completely absent [7,11]. Given the importance of the SSA as the blood supply not only to the rotator cuff muscles but also to the acromioclavicular and glenohumeral joints and the clavicle, knowledge of the variations of the SSA is of utmost importance to clinicians, particularly in the preparation and completion of rotator cuff and shoulder surgeries. DSA has also been shown to have variant origins from TCT, TCA, the second part of SA, and the AA [12-15]. Variability also exists in the structures that the DSA supplies besides its normal blood supply to the posterior back muscles [11]. Understanding of variations in DSA and SSA is also of vital importance because of their participation in scapular anastomosis [1]. In this case study, we describe anatomical variations in the origin and course of the branches of SA (SSA and DSA) with potential clinical implications of these findings.

This article was previously presented as a meeting abstract at the following conferences: (1) the American Association for Anatomy Annual Meeting on March 31, 2025; (2) the American Association for Anatomy Regional Meeting on October 7, 2023; and (3) the Annual Clinical Assembly of Osteopathic Surgeons on September 22, 2023.

## Case presentation

During routine dissection of the right neck of a 75-year-old Caucasian male donor, an unusual origin and course of the SSA was observed. The donor for the case report was obtained through the Willed Body Program at the University of Texas Health at San Antonio for the purposes of medical education and research. All donations made to the program are registered and cared for by guidelines and statutes laid out by the State of Texas. Skin, subcutaneous fat, and superficial fascia of the anterior and lateral regions of the neck were first removed. The sternocleidomastoid muscle was reflected to visualize the underlying musculature and neurovasculature. Utmost care was taken to preserve the origin and course of the branches of SA. Posterior back muscles (trapezius, rhomboid major, and minor muscles) were reflected to follow the arterial supply to these muscles. Following the discovery of the variations on the right side of the donor, the cervical region and posterior back muscles were dissected on the left side of the donor body to determine if the variations are bilateral or unilateral.

Anatomical variation of the SSA

**Figure 1:**
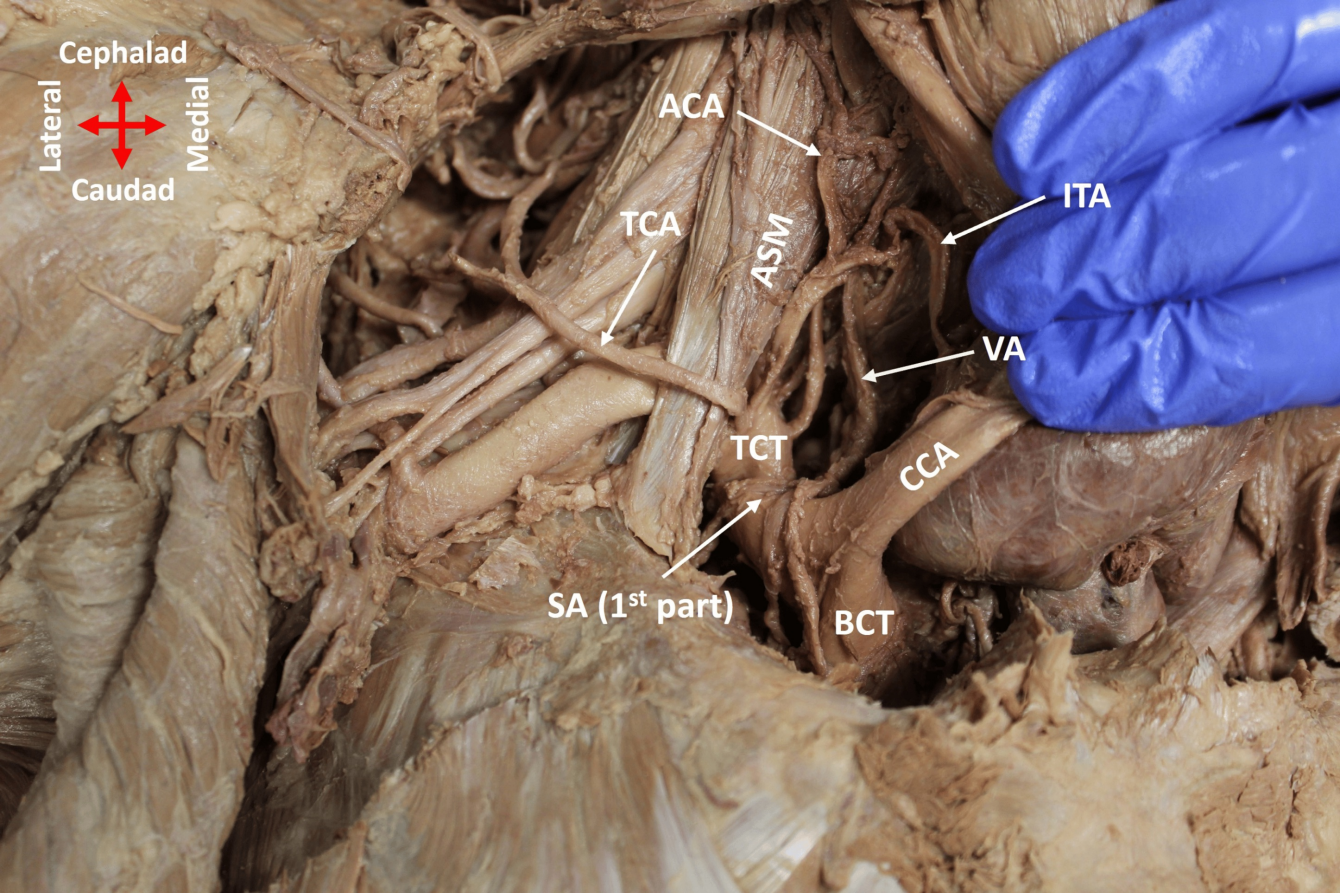
SSA did not arise from the TCT on the right side of the body Only TCA, ITA, and ACA emerged from the TCT in the first part of the SA. CCA was retracted to the left to show the TCT branches. ACA: ascending cervical artery; ASM: anterior scalene muscle; BCT: brachiocephalic trunk; CCA: common carotid artery; ITA: inferior thyroid artery; SA: subclavian artery; SSA: suprascapular artery; TCA: transverse cervical artery; TCT: thyrocervical trunk; VA: vertebral artery

**Figure 2:**
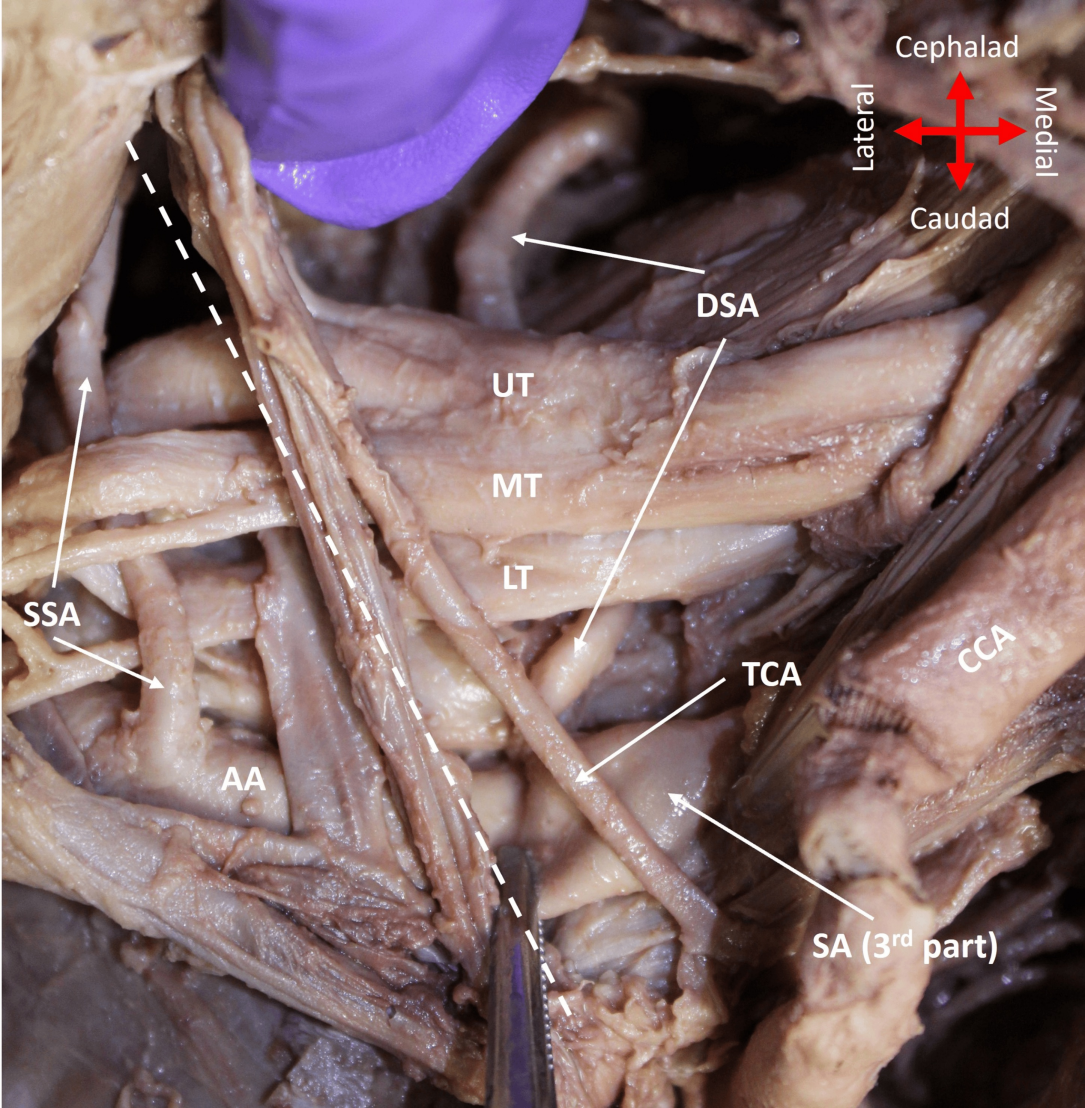
Direct origin of the SSA from the first part of AA on the right side of the body SSA emerged directly from the AA, passed over the LT, and continued obliquely between the contributions of the UT and MT to the lateral cord. The dashed line shows the path of the first rib. AA: axillary artery; CCA: common carotid artery; DSA: dorsal scapular artery; LT: lower trunk; MT: middle trunk; SA: subclavian artery; SSA: suprascapular artery; TCA: transverse cervical artery; UT: upper trunk

**Figure 3:**
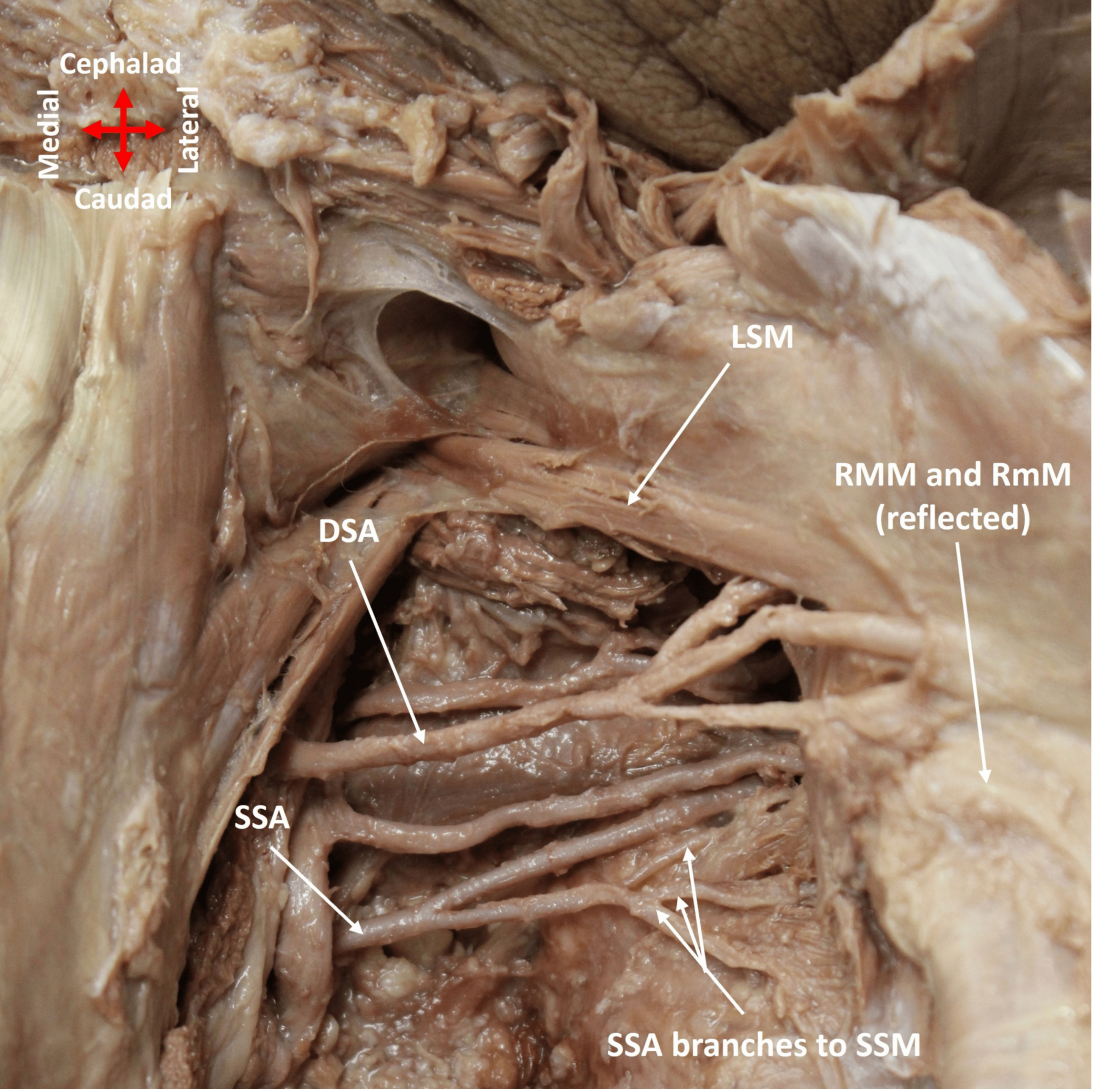
SSA provides blood supply to the subscapularis muscle Posterior view of the right scapular region showing the course of the SSA and DSA. SSA is divided into two branches, one of which is divided further into three smaller branches to supply the subscapularis muscle. DSA: dorsal scapular artery; LSM: levator scapulae muscle; RMM: rhomboid major muscle; RmM: rhomboid minor muscle; SSA: suprascapular artery; SSM: subscapularis muscle

The TCT had three of the four normal branches on the right side of the neck: TCA, ITA, and ACA (Figure 1). The SSA did not arise from the TCT; instead, it emerged directly from the first part of the AA, which was determined by its origin past the lateral border of the first rib, where the SA transitions to the AA (Figure 2). The SSA passed over the lower trunk and continued obliquely between the contributions of the upper and middle trunks to the lateral cord of the brachial plexus (BP) (Figure 2). It coursed posteriorly, where it divided into two branches (Figure 3). While one branch continued to the supraspinous fossa, the second branch split into three smaller branches to supply the subscapularis muscle (Figure 3).

Anatomical variation of the DSA

**Figure 4:**
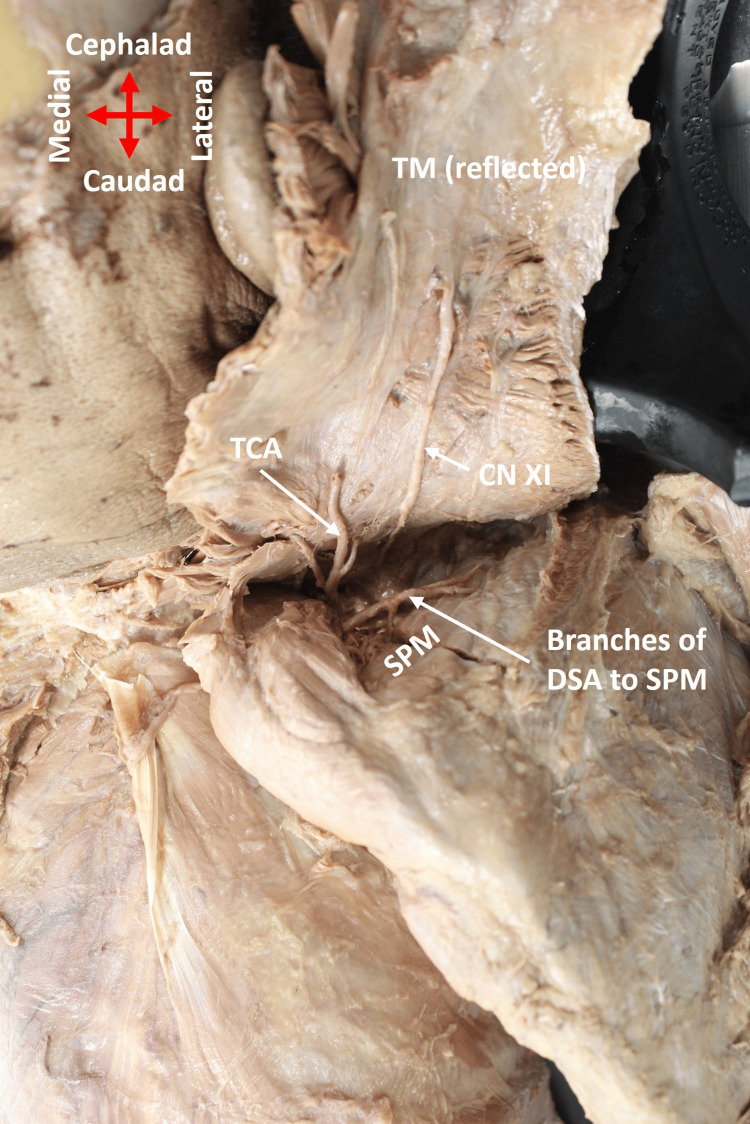
Branches of DSA provide blood supply to the supraspinatus muscle The trapezius muscle was reflected superiorly to show the vasculature and muscles. CN XI: cranial nerve XI (accessory nerve); DSA: dorsal scapular artery; SPM: supraspinatus muscle; TCA: transverse cervical artery; TM: trapezius muscle

The DSA emerged normally from the third part of the SA lateral to the ASM (Figure 2). Thereafter, it coursed posteriorly, deep to the upper and middle trunks of the BP. Importantly, in addition to its normal blood supply to the rhomboid and levator scapulae muscles (Figure 3), DSA also provided additional branches to the supraspinatus muscle (Figure 4).

No such variations were observed on the left side of the donor body.

## Discussion

The SSA typically originates from TCT and takes a lateral course superficial to the ASM, phrenic nerve, and the BP to reach the supraspinous fossa; however, some cadaveric studies have reported variations in the origin and course of SSA or its complete absence [3-7,9-11,16]. Weiglein et al. investigated 498 neck-halves across three sites in Austria and Germany and showed SSA to originate from the TCT (27%), SA (12%), and the ITHA (11%) [10]. Additionally, the SSA emerged from the cervico-scapular trunk in 22% of the cases, the dorso-scapular trunk in 4% of the cases, and the cervico-dorso-scapular trunk in 24% of the cases [10]. In another cadaveric study, Pyrgakis et al. showed SSA to emerge from the third part of the SA in only one of the 62 necks studied (1.6%) before coursing toward the scapular region, superficial to the BP [5]. Takafuji et al. demonstrated that the SSA branches directly from the SA in 26% of cases (37/144 sides), whereas in 2.8% of the cases (4/144 sides) it was completely absent [7]. We made a similar observation in a recent study where SSA was unilaterally absent in an 87-year-old Caucasian male donor [11]. An interesting case report by Alexander et al. found the subscapular artery to be the origin of SSA, which then took a long and torturous course to enter the supraspinous fossa [16]. Some cadaveric reports have also demonstrated the variable origin of the SSA from the first part of the AA [3-4,9], similar to what we found in this study. These studies further show that when SSA originates from AA, it courses laterally through parts of the BP instead of passing superficial to it. Piagkou et al. showed SSA to course between the lateral and medial cords of the BP in a 91-year-old male donor [9], whereas another study in a female of North Indian origin showed SSA to cross anteriorly by the lateral cord [3]. Tsakotos et al. recently showed SSA to course through the anterior and posterior division of C5, 6, and 7 spinal nerves’ fusion in an 80-year-old female donor of Greek origin [4]. Dargaud et al. explored the relationship of SSA and BP in cadavers of European origin (100 dissections) and found that in 28% of cases, the SSA passed between the superior and middle trunks of BP, whereas in 1% of cases, the vessel passed behind the BP [17]; however, this study did not focus on where the SSA originated. In our study, the SSA traversed to the supraspinous fossa between the contributions of the upper and middle trunks to the lateral cord of the BP. Additionally, the SSA provided blood supply to the subscapularis muscle, a finding that was also reported in a recent study by Tsakotos et al. [4].

DSA normally arises from the third part of the SA but can have variable origins from the TCT, the second part of the SA, TCA, and AA [12-15]. In this study, the DSA had a normal origin and coursed posteriorly through the BP to provide blood supply to the levator scapular and rhomboid muscles. However, it additionally provided branches to the supraspinatus muscle. We made a similar observation in a previous cadaveric study in which TCA and SSA were both absent [11]. To the best of our knowledge, these are the only two studies that have documented DSA as a source of blood supply to the supraspinatus muscle.

The SSA irrigates the rotator cuff in general and the supraspinatus/infraspinatus muscles, clavicle, and shoulder joint in particular. Hence, SSA variants may affect treatments involving the rotator cuff and clavicle fractures. Moreover, during radical and modified neck surgeries, the SSA must be identified and ligated. Therefore, variation in the origin and course of the SSA, such as that seen in our case, takes on great importance. There is also concern in regard to rotator cuff surgeries, which involve a tear in the subscapularis, infraspinatus, teres minor, and/or supraspinatus muscles. Approximately 30% of the population over 60 reports a rotator cuff tear, with the prevalence increasing significantly in those over 80, to 62% [18]. Postoperative outcomes are generally positive, with 65.7% of patients reporting good and excellent results [19]. These surgeries are often performed under the assumption that the patient exhibits normal vascular anatomy. If rotator cuff surgery were to be performed on a patient with this variation in blood supply, an artery could potentially be compromised. The unusual branching pattern at the origin of these arteries could also have impacted a variety of less common surgeries with an anterior cervical approach. If silent variations, such as the ones seen in the donor, were present, these surgical approaches could potentially damage the unique branching pattern, leading to hemorrhage at the site of the injury and necrosis in the termination points of these variations.

Generally, nonpathological causes of anatomical variation are not well defined or understood, as individuals with these variations rarely report symptoms during life. However, in this donor’s case, we can make some general assumptions about the potential origin of the observed variations. Embryologically, the proximal portion of the right SA arises from the fourth aortic arch, and the more distal portion is formed by the right seventh intersegmental artery. However, the left SA is only formed by the seventh intersegmental artery. This difference in origin could be an explanation for the unilateral variations noted in this individual. One of the observed variations, the SSA, involves a displacement of the origin from the proximal portion to the distal portion of the SA. This displacement could be due to a failure of the right 4th aortic arch to fully form all normal branches.

## Conclusions

This study describes the variable origin of the SSA from the AA, its course through the BP, and its branches supplying the subscapularis muscle. In addition, the study also reports DSA as a source of blood supply to the supraspinatus muscle. Knowledge of these vascular anomalies may be important for vascular and orthopedic surgeons during procedures in the shoulder region, such as the repair of rotator cuff injuries, scapulectomy, and clavicle fractures, and for the education of medical students. Future directions include population-based cadaver and angiographic studies to determine the occurrence of these clinically relevant vascular anomalies.
